# Mechanics of sucking: comparison between bottle feeding and breastfeeding

**DOI:** 10.1186/1471-2431-10-6

**Published:** 2010-02-11

**Authors:** Angel Moral, Ignasi Bolibar, Gloria Seguranyes, Josep M Ustrell, Gloria Sebastiá, Cristina Martínez-Barba, Jose Ríos

**Affiliations:** 1 Department of Pediatrics, Neonatology Section, Hospital Universitario Mútua de Terrassa, Barcelona, Spain; 2 Department of Pediatrics, Hospital Sant Joan de Dèu de Martorell, Barcelona, Spain; 3 CIBER de Investigación Biomédica en Red en Enfermedades Raras (CIBERER), Barcelona, Spain; 4 Department of Clinical Epidemiology and Public Health, Hospital de la Santa Creu i Sant Pau, Autonomous University of Barcelona, Barcelona, Spain; 5 CIBER de Epidemiología y Salud Pública (CIBERESP), Barcelona, Spain; 6 Midwifery Teaching Unit of Catalonia, Nursing School, University of Barcelona, Barcelona, Spain; 7 Faculty of Odontology, University of Barcelona, Barcelona, Spain; 8 Institut Clinic de Ginecologia Obstetricia i Neonatologia (ICGON), Corporació Sanitaria de l*Hospital Clínic, Barcelona, Spain; 9 Primary Health Care Center Vinyets, Catalan Institute of Health, Sant Boi de Llobregat, Barcelona, Spain; 10 Unit of Biostatistics, Faculty of Medicine, Autonomous University of Barcelona, Barcelona, Spain

## Abstract

**Background:**

There is very little evidence of the similarity of the mechanics of maternal and bottle feeding. We assessed the mechanics of sucking in exclusive breastfeeding, exclusive bottle feeding, and mixed feeding. The hypothesis established was that physiological pattern for suckling movements differ depending on the type of feeding. According to this hypothesis, babies with breastfeeding have suckling movements at the breast that are different from the movements of suckling a teat of babies fed with bottle. Children with mixed feeding mix both types of suckling movements.

**Methods:**

Cross-sectional study of infants aged 21-28 days with only maternal feeding or bottle feeding (234 mother-infant pairs), and a randomized open cross-over field trial in newborns aged 21-28 days and babies aged 3-5 months with mixed feeding (125 mother-infant pairs). Primary outcome measures were sucks and pauses.

**Results:**

Infants aged 21-28 days exclusively bottle-fed showed fewer sucks and the same number of pauses but of longer duration compared to breastfeeding. In mixed feeding, bottle feeding compared to breastfeeding showed the same number of sucks but fewer and shorter pauses, both at 21-28 days and at 3-5 months. The mean number of breastfeedings in a day (in the mixed feed group) was 5.83 ± 1.93 at 21-28 days and 4.42 ± 1.67 at 3-5 months. In the equivalence analysis of the mixed feed group, the 95% confidence interval for bottle feeding/breastfeeding ratio laid outside the range of equivalence, indicating 5.9-8.7% fewer suction movements, and fewer pauses, and shorter duration of them in bottle feeding compared with breastfeeding.

**Conclusions:**

The mechanics of sucking in mixed feeding lay outside the range of equivalence comparing bottle feeding with breastfeeding, although differences were small. Children with mixed feeding would mix both types of sucking movements (breastfeeding and bottle feeding) during the learning stage and adopt their own pattern.

## Background

Feeding in the neonatal period is a complex activity demanding efficient coordination between the rhythmic processes of suck, swallowing, and respiration [1]. Several factors can influence the rhythm with which babies perform the sucking runs and pauses, including age, hunger, baby's mouth position on the breast, sucking time and pressure, fatigue and satiation, and milk flow. There is some debate regarding the best bottle teat to be used to enhance the bottle-feeding performance, particularly in very low birth weight infants [2]. Ultrasound techniques have been used to document in vivo the anatomic characteristics of the human nipple during breast-feeding and to visualize artificial teats during sucking [3,4]. Although a number of studies have assessed the differences in sucking patterns between preterm and full-term infants [5-9] little research has been carried out addressing mechanics of nutritive sucking during bottle feeding with different teats [10-13]. A wide variability in performance has been observed not only between different types of term and preterm teats but also within the same type [11]. One limitation of these feeding studies concerns to the lack of comparison with breastfed babies.

Environmental conditions including habits of nonnutritive sucking, such as thumb sucking and the use of a pacifier, or bottle feeding have been claimed to eventually contribute to dental malocclusion [14-16]. Physiological teats were designed to maintain morphologic and physiologic functional characteristics of the nursing infant during bottle feeding. Sucking patterns studied in breastfed or bottle feed babies with these teats showed similar mechanics of nutritive sucking related to sucking movements, pauses, and sucking pressures [17]. Another claim against bottle feeding is nipple confusion which makes the babies preferences for the teat flow that produce the most milk with the least effort instead the nipple flow [18].

The aim of the present study was to assess mechanics of feeding movements in exclusive breastfeeding, bottle feeding, and mixed feeding. The hypothesis established was that physiological pattern for the suckling movements in a child differ depending on the type of feeding being received. According to this hypothesis, babies with breastfeeding have suckling movements at the breast that are different from the movements of suckling a teat of babies fed with bottle. Children with mixed feeding mix both types of suckling movements during the learning stage and adopt their own pattern.

## Methods

### Study Design

This was a descriptive, cross-sectional study of mechanical patterns of infant feeding with exclusive breastfeeding, bottle feeding and mixed feeding. In the case of mixed feeding, a randomized open cross-over field trial was carried out to assess the equivalence of the mechanical patterns between breastfeeding and bottle feeding. In the cross-over trial, the order of the type of feeding was decided at random. Two groups of babies were studied: newborn aged 21 to 28 days and infants between 3 and 5 months of age. In the youngest group with mixed feeding and in order to reduce the heterogeneity in the mechanics of each feeding session, two successive observations were made for each type of feeding, with a total of four measurements for each random sequence. The infant group had two observations, also in a random sequence. A computer-generated randomization list was prepared in advance for each center, one for the newborns from 21-28 days of age and another one for the infants of 3-5 months of age. The random assignment was centrally obtained from the coordinating center through a telephone call.

### Subjects

The subjects were term infants born at ≥ 37 wk gestational age with a birth weight ≥ 2500 g whose mothers spontaneously attended mother-child health care centers after childbirth in Barcelona, Spain. A total of 13 centers, public or private, participated in the study.

Newborns (21-28 days old) and infants (3-5 months old) were eligible if one of these conditions were satisfied: 1) exclusive breast-feeding, 2) exclusive bottle-feeding, 3) mixed feeding with an adaptation period of at least 2 weeks in both types of feeding to ensure their correct use. The mothers' decision about the type of feeding was always respected and the inclusion in the study did not influence on this decision. Mixed feeding was considered when the baby combined breast milk and formula in the same or alternative feeding. Mother-infant pairs were recruited between May 2004 and June 2006. Those with pre-existing feeding problems, including the following conditions were excluded: congenital anomalies of the maxilla-facial and upper digestive tract, upper airway dimorphism, upper infection during the previous week or at the time of the study, systemic or weakening diseases, suckling or swallowing disorders, problems of the mother-child relationship, latex allergy, lack of habituation to the studied type of feeding, and maternal contraindications for breastfeeding, including HIV infection, hepatitis, type 1 herpes simplex virus, flat, inverted, or cracked nipples, and mastitis. The study protocol was approved by the ethics committees of the participating centers, and written informed consent was obtained from the parents.

### Phases of the Study

Study measurements were scheduled with a maximal number of observations of 2/day, making sure that the child had not been fed in the previous 2 hours. The observations were planned as close as possible, and at the same hour of the day, to minimize the period effect (differences in the feeding measurement conditions for each period). The study days were to be separated by a maximum interval of 7 days. As the newborns of 21-28 days of age with exclusive breastfeeding or bottle feeding required a single feeding measure, one study session was scheduled for them. Newborns of 21-28 days of age with mixed feeding required four measurements; therefore, four study sessions were scheduled. In infants of 3-5 months of age, who required two measurements, two feeding sessions were scheduled. The place for taking the feeding measurements was in the mother-child care centre, or at mother's home.

On the day of the study, correct performance of breastfeeding, bottle feeding, or mixed feeding was firstly assessed. Correct use of mixed feeding required a minimum of two breastfeeding intakes a day in the 2 weeks before the day of the study, and that the child had been fed at least two times a day and a maximum of four times a day with bottle in the same period. Otherwise the study was postponed. Then, the sequence of the type of feeding was randomized in the group of mother-infants with mixed feeding.

### Feeding Sessions

All measurements were made by registered nurses specifically trained for the study, and under the same conditions in all sites. Nurses were trained by only one person (CMB) and supervised in at least 6 sessions to ensure that observations were made alike. The length of the feeding observation was identical for all infants in the same age group. It was 10 min for infants of 21-28 days and 15 min for the 3-5 month group. A feeding session was considered suitable for the inclusion in the study when the milk sucking-extraction movements had been recorded for at least 5 min. At the beginning of the feeding sessions, special care was taken by the monitor nurse to ensure correct fulfillment of the feeding conditions, including a comfortable environment and, more importantly, adequate position of the baby's head and trunk for proper attachment and optimal positioning of the teat or the nipple and areola into the infant's mouth.

Infants were fed either formula or breast milk, and were allowed to feed at their own pace. None of the mothers fed her child with breast milk in a bottle. Infant formulas according to the European Society for Paediatric Gastroenterology, Hepatology and Nutrition guidelines [19] and adequate to the age of the child were used. The amount provided in the feeding bottle was 120 mL for newborns of 21-28 days of age and 180 mL for infants aged 3-5 months. In order to obtain more homogeneity in the feeding measurements, the NUK First Choice latex teat (MAPA GmbH, Zeven, Germany) was used by all the bottle fed babies. Teats were provided to the participating mothers when they started the bottle feeding (newborns of 21-28 days of age) or if they were already giving mixed feeding (infants of 3-5 months of age) when they joined to the study. The medium-size hole was used in all cases. All babies used the same teat at home. The size of the feeding bottle was 150 mL for infants of 21-28 days and 300 mL for the 3-5 month group.

### Outcome Measures

The number of sucks per minute over the 10 or 15 min feeding observation in babies of 21-28 days and 3-5 months of age, respectively, was used as the primary outcome of the oral feeding performance. Sucks were counted by direct observation of the movements of the jaw (masseter muscle). Other instrumental non-invasive methods (like ultrasounds) were discarded because its use might influence the mother-child interaction during feeding, produce a distraction of the baby and modify the actual sucking movements. Pauses-related data were also recorded as elements of the mechanics of suction. These included number of pauses (no sucking > 1 s), duration of pauses, pauses per minute and duration of pauses per minute of feeding. The feeding time (interval between the first and last suction movements of feeding) was monitored with a mechanical stopwatch. During each study session, the nurse said aloud all the outcomes (start of the feeding, number of suckling movements at each minute, beginning and end of each pause, and end of the last suckling movement) and recorded them in an audiotape. Afterwards, the nurse translated the data to the case report form. This method allowed an accurate observation of the events per minute of the session.

### Data Analysis

Data reported in the study of Usadel [17] were used for calculating the sample size, which was estimated to determine whether in babies with mixed feeding, the use of the teat was equally efficient, in terms of sucks/min, as breastfeeding. The formula of Liu and Chow [20] for equivalence studies was applied. With 62 assessable subjects per group (124 in total) there was a power of 80% for determination of the equivalence defined by a test/reference ratio [Average_bottle feeding_/Average_breastfeeding_] and 95% confidence interval (CI) of the main variable (sucks/min) within the delta margin of tolerability of ± 5%, assuming a two-sided type I error of 5% and a maximum coefficient of variation (CV) of 12% derived from the residual variance of the analysis of variance (ANOVA) after log-transformation. A total number of 372 children was required, 248 newborns of 21-28 days and 124 infants of 3-5 months of age, with a total number of 868 measurements.

According to a delta of 5%, the number of sucks/min for breastfeeding and bottle feeding, respectively, would be equivalent, with the 95% CI for the log transformed bottle feeding/breastfeeding ratio and their 95%CI within the equivalence limits of 95.0% to 105.3%. Analyses of variance for cross-over design were performed for the estimated values of ratios for log-transformed values of the three independent variables. For these models a mixed random effect was considered with the subject nested into sequence. Period and type of feeding effects were also assessed. Winnonlin ver 5.0.1 module of linear mixed effects model was used. The Statistical Analysis Systems (SAS Institute, Cary, NC) statistical software package was used for the analysis of the rest of data. A two-sided P value ≤ 0.05 was considered statistically significant. To evaluate mean differences between or within groups, unpaired t-test or the appropriated paired test were used for the sucking parameters.

## Results

### Study Population

Of a total of 463 mother-infant pairs eventually recruited for the study, 104 were excluded for the following reasons: birth weight <2500 g (n = 4); cleft palate (n = 1); refusal to participate (n = 51); discontinuation of breastfeeding within the 3-month period after childbirth (n = 18); use of a different teat of the study (n = 15); diseases in the mother (n = 4); retracted nipple (n = 2); and other reasons (n = 9). Therefore, the study population consisted of 359 mother-infant pairs, the distribution of which according to the type of feeding is shown in Table 1. The main characteristics of the mother-pairs included in the study are summarized in Table 2. All groups were comparable with regard to the age of the mothers, sex of the infants, gestational age, birth weight, and time elapsed between the last feeding and the first feeding session in which suck parameters were recorded. The overall percentage of twin pregnancies was 8%, and ranged between 0% and 15%, depending on the subgroup of the study. In the mixed feeding group, the daily mean number of breastfeedings and bottle feedings recorded during the previous week was also comparable.

**Table 1:** Number of infants in the study according to the type of feeding.

Ages and Type of Feeding	Number of Infants		Number of Feeding Measures	
	Required	Included (%)	Required	Performed (%)
**21-28 days**				
Breastfeeding only	62	62 (100)	62	62 (100)
Bottle feeding only	62	62 (100)	62	62 (100)
Mixed feeding				
Randomization sequence				
BF-BF-FF-FF	124	110 (88.7)	496	440 (88.7)
FF-FF-BF-BF				
				
**3-5 months**				
Mixed feeding				
Randomization sequence	124	125 (100.8)	248	250 (100.8)
BF - FF FF - BF				
				
**Total**	372	359 (96.5)	868	814 (93.8)

BF: natural feeding (breastfeeding); FF: formula feeding (bottle feeding).

**Table 2:** Characteristics of the mother-infant pairs included in the study.

**Data**	**Age Groups and Type of Feeding**			
	**21-28 days**			**3-5 months**
		
	**Breastfeeding only** **(n = 62)**	**Bottle feeding only** **(n = 62)**	**Mixed feeding** **(n = 110)**	**Mixed feeding** **(n = 125)**
Mother, age, years, mean ± SD	32.0 ± 4.8	31.9 ± 4.6	32.7 ± 5.4	32.5 ± 4.5
Type of pregnancy, n (%)				
Single	62 (100)	54 (93.1)	80 (85.1)	104 (90.4)
Twins	0 (0)	4 (6.9)	14 (14.9)^‡^	11 (9.6)^§^
Sex of the infant, no, (%)				
Male	35 (56.5)	30 (49.2)	54 (49.5)	64 (51.2)
Female	27 (43.5)	31 (50.8)	55 (50.5)	61 (48.8)
Gestational age, wk, mean ± SD	39.8 ± 1.2	39.4 ± 1.6	39.0 ± 1.3	39.4 ± 1.4
Birth weight, g, mean ± SD	3301.6 ± 349.2	3238.8 ± 501.9	3214.2 ± 429.8	3241.1 ± 451.4
Infant's age on the first feeding measure, days, mean ± SD	25.4 ± 2.1	24.5 ± 2.6	24.5 ± 3.6	120.8 ± 52.9
Feedings per day in the last week				
Breastfeeding, mean ± SD	NA	NA	5.8 ± 1.9	4.4 ± 1.7
Bottle feeding, mean ± SD	NA	NA	5.0 ± 2.2	3.7 ± 1.6
Time since the last feeding, h, mean ± SD	2.8 ± 0.7	3.2 ± 0.6	3.1 ± 0.6* 3.1 ± 0.7^†^	3.5 ± 1.5* 3.4 ± 1.1^†^

^‡^Data missing in 2 newborns; ^§^One twin did not enter the study.

*Breast feedings; ^†^bottle feedings.

### Sucking Parameters

Results of sucking parameters are shown in Table S3 (see additional file 1). The mean number of sucks/minute recorded for the study period in newborns aged 21-28 days was significantly higher among breast fed but only when the groups of exclusive breastfeeding and bottle feeding were compared. In mix fed infants, either aged 21-28 days or 3-5 months, the differences in the mean number of sucks/minute for breastfeeding and bottle feeding during the study period were not statistically significant.

In the group of newborns aged 21-28 days, exclusive bottle feeding was characterized by a no different number of pauses/minute than exclusive breastfeeding but of significantly longer duration. In mixed feeding, bottle feeding showed significantly fewer pauses/minute, and of shorter duration/minute, as compared with breast feeding. The same pattern was observed in infants with mixed feeding aged 3-5 months.

**Figure 1:**
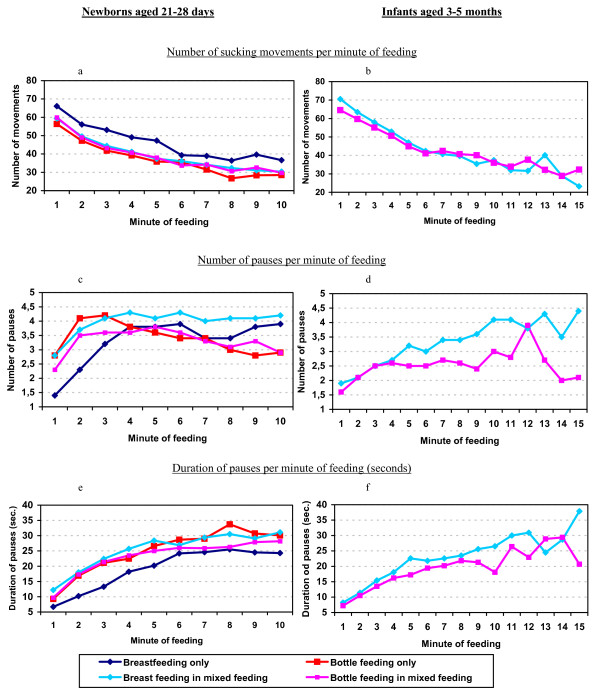
**Evolution of mechanical parameters per minute of feeding**.

The mean number of sucks in exclusive breastfed newborns was the highest in each studied minute, whereas it was the lowest in exclusive bottle fed newborns; mixed fed newborns showed an intermediate number of sucks, either in a breast feeding or in a bottle feeding (fig. 1a). Mixed fed infants (age 3-5 months) showed also a progressively lowering trend, which was similar either in breast feeding and bottle feeding (fig. 1b). The evolution of the number of pauses per minute of feeding in newborns showed a two phases trend in all types of feeding, with an initial increase of the pauses per minute from 0 to a maximum of slightly more than 4 pauses/minute, and then it was maintained until the end of the study period, but only in the group of breast feeding (fig. 1c). In the bottle fed newborns, the second phase showed a decreasing trend. In infants, the number of pauses per minute followed a constantly increasing trend, which was superior in the breast feeding observation (fig. 1d). The evolution of the number of the duration of pauses per minute is shown in fig. 1e. In newborns, the duration increased from minute 1 to minute 6, and then remained fairly constant. In infants, however, the increase of pause duration was more constant in all the study period, but bottle feeding presented a shorter duration in most minutes of feeding (fig. 1f).

### Equivalence of Sucking Patterns in Mixed Feeding

In infants of 21-28 days of age, the bottle feeding/breast feeding ratio (95% CI) for the total number of suck movements was 91.3% (95% CI 85.2-97.9) and for infants aged 3-5 months 94.1% (95% CI 86.3-102.6). This means that, overall, there were 8.7% and 5.9% fewer suction movements in bottle fed newborn and infants, respectively. The ratio for the total number of pauses was 73.9% (95% CI 67.1-81.3) and 82.2% (95% CI 71.2-95.0) for the groups of 21-28 days and 3-5 months of age, respectively. The corresponding values for total duration of pauses were 77.1% (70.5-84.3) in infants aged 21-28 days and 75.3% (95% CI 62.4-91.0) in babies 3-5 months of age. In all cases, 95% CI of the bottle feeding/breastfeeding ratio lay outside the range of equivalence of 95.0% to 105.3%.

## Discussion

This study adds information on mechanics of nutritive sucking of breastfeeding, bottle feeding and mixed feeding in newborns aged 21-28 days and in 3-5-month-old babies. When we looked at the number of sucking movements in the exclusive breastfeeding group and the bottle feeding group, we saw a statically significant difference. In the mixed feed group of newborns, the number of sucking movements per minute was in the middle of both exclusive feeding groups.

The analysis of equivalence of sucking parameters between breastfeeding and bottle feeding in mix fed infants demonstrated that newborns with a mixed feeding made an 8.7% less of sucking movements when they were fed with a bottle compared with the sucking movements they did on the breast. In the infant group (3-5 month old), the difference was a 5.9% less sucking movement on the bottle compared with the breast. Both of these differences lie outside of the tolerability margin of ± 5% defined in this study. However, the magnitude of the differences in terms of mean suction movements was small. The present results are reinforced by the randomized cross-over design, in which each infant served as his/her own control. Our findings are also supported by a large number of observations, 440 feeding measurements recorded in 110 mother-infant pairs for the age group of 21-28 days and 250 in the 125 mother-infant pair for the age group of 3-5 months. In addition, a comparative study of breastfeeding and bottle feeding using a prospective randomized parallel design, in which the mother is assigned to one specific type of feeding, is difficult to be carried out for ethical reasons.

Different studies have shown that sucking profiles of the preterm infant are significantly different from the full-term infant [5,12,21] but as far as we are aware, no previous studies except for the study of Usadel [17] in the 50's have been published reporting a comparison of mechanics of nutritive sucking in full term healthy infants using breast milk or bottle feeding, or combined feeding by breast milk and formula. For this reason, the present findings cannot be discussed in the light of data of published by other authors. Qureshi et al. [7] quantified rhythmic suckle feeding in 16 infants with bottle feeding at 1 to 4 days of age and again at 1 month and observed that suck rate increased significantly from 55/min in the immediate postnatal period to 70/min by the end of the first month. In our study, the suck rate was lesser in our newborn group with mixed feeding (41-42/min). On the other hand, in a study of how sucking performance changes from non-nutritive sucking (NNS) to nutritive sucking (NS), Mizuno and Ueda [22] also found that bottle feeding differs from breastfeeding. The sucking pressure at breast was higher during NNS compared with NS, whereas with a bottle the sucking pressure was lower during NNS compared with NS. These authors explained the difference because of the milk ejection reflex. However, sucking performance in terms of pressure measurements was not assessed in our study. On the other hand, feeding volume at breastfeeding was not measured. Feeding children with breast milk in a bottle was not allowed nor the use any method that might influence the natural mother-child interaction during feeding. In addition, the difference of weight of intake does not equal the caloric intake. The infant formula has a homogeneous constitution, but this is not the case with breast milk. The difficulties in the conversion of the volume of the intake to a comparable measure between breastfeeding and bottle feeding led us to the no inclusion of this variable in the study protocol. Additionally, we did not use any method that could interfere in the natural mother-child interaction during feeding; therefore, nutritive sucking cannot be differentiated from non-nutritive sucking. However, the number of sucks per minute over the 10 or 15 min feeding sessions was the primary outcome variable of the study.

In infants of 21-28 days of age using bottle feeding alone, mechanics of sucking compared to exclusive breastfeeding is characterized by fewer suck movements and the same number of pauses but of longer duration.

In mixed feeding, bottle feeding, compared to breastfeeding, showed the same number of sucks per minute (P = 0.577 at 21-28 days and P = 0.094 at 3-5 months). However, as seen before, the absence of statistically significant differences didn't mean that both types of feeding were equivalent. Similarly, there were fewer and shorter pauses per minute in bottle feeding compared to breastfeeding, both at 21-28 days and at 3-5 months. The lesser number of pauses, although statistically significant, was small (difference of means 0.6 and 0.4 pauses/min in newborn and infant groups, respectively). In addition, the lesser duration of the pauses was also small (difference of means 3.0 and 2.6 s/min in newborn and infant groups, respectively). In all study groups, a progressive decrease in the number of sucks/min accompanied by an increase in the number and duration of pauses/min along the feeding period was observed. In general, for the small pause differences observed between natural and bottle feeding in the mixed feeding group the changes in the density and caloric content of the breast milk should be taken into consideration. Their progressive increase during breastfeeding would contribute to a greater sensation of satiety in the baby and could explain the small increase observed in the duration of the pauses. The important reasons of the pauses in breastfeeding are the milk flow pattern and the baby, whereas in bottle feeding depends on the mother. The potential clinical relevance of these small differences on grounds of development of the child was not assessed in the present study. To this end, we would need a prospective, long-term, follow-up study, which escapes of the scope of our current objectives. Other future studies could include methods of measurement that are more objective than the direct observation of the jaw movements and that could allow a more accurate differentiation between nutritive and non-nutritive sucking movements. Ideally, these methods should not be invasive, not interfere with the baby and the feeding, and sufficiently efficient to be used in large population sample studies. The fact that it was not requested to the mother to explain how long they had been mixed feeding for and the reason of the mixed feeding may be considered a limitation of the study. However, our sample of study was composed of non-institutionalized, healthy mothers, without problems for breastfeeding and who used mixed feeding according to their own decision. Finally, although the study sample of mothers and babies was substantial, we could not fully achieve the predefined sample size. Nevertheless, the number of required infants and measures were very close to that finally included in the study (97% and 94% respectively). Implications of these slight discrepancies are minimum, even though the small differences observed in the equivalence analysis, and do not justify a change in the conclusions.

## Conclusions

The babies with exclusive breastfeeding show a nutritive sucking pattern different from the babies with exclusive bottle feeding. In the newborns and infants with mixed feeding, the equivalence analysis showed that fewer suction movements and pauses, and shorter duration of pauses, occurred in bottle feeding compared with breastfeeding. The mechanics of nutritive sucking of breastfeeding and bottle feeding in mixfed infants is not the same, but the observed differences were small. Children with mixed feeding would mix both types of sucking movements (breastfeeding and bottle feeding) during the learning stage and adopt their own pattern.

## Abbreviations

ANOVA: analysis of variance; CI: confidence interval.

## Competing interests

Roche Diagnostics, S.L. (Sant Cugat del Vallés, Barcelona, Spain) sponsored the study costs. Study design, supervision of the field work and data collection, analysis and interpretation of data, writing of the report and decision to submit the paper for publication were done by the authors who receive an honorarium from the sponsor.

Any author has non-financial competing interests.

## Authors' contributions

All authors have made substantial contributions to the study, have been involved in drafting the manuscript or revising it critically for important intellectual content. All authors read and approved the final manuscript. Each author takes full responsibility for the reported research. AM, IB, G Seguranyes, JMU, and G Sebastiá made up the Scientific Management Committee, composed by one paediatrician, two nurse midwifes, one epidemiologist, and one orthodontist. They defined the conception and design of the study, made the literature review, coordinated the study, assessed and interpreted the results and contributed to writing of the paper. CM-B: was the monitor-supervisor and coordinated the field work and the acquisition of data. JR: was the statistician of the Centre for Data Processing and Analysis. He performed the sample size calculation, statistical analysis of data and interpretation of results.

## Pre-publication history

The pre-publication history for this paper can be accessed here:

http://www.biomedcentral.com/1471-2431/10/6/prepub

## Supplementary Material

Additional file 1 **Table S3**. Results of sucking parameters. Click here for file
