# Supplementary material for: Mechanics of sucking: comparison between bottle feeding and breastfeeding
Source: BMC Pediatr. 2010 Feb 11;10:6. doi: 10.1186/1471-2431-10-6 (PMC2837866; doi:10.1186/1471-2431-10-6)
Supplement: Additional file 1 — Table S3. Results of sucking parameters. [file 1471-2431-10-6-S1.PDF]

TABLE S3. Results of sucking parameters.

Data	21-28 Days of Age			21-28 Days of Age and Mixed Feeding			3-5 Months of Age and Mixed Feeding		
	Breastfeeding only	Bottle feeding only	*P* value	Breastfeeding	Bottle feeding	*P* value	Breastfeeding	Bottle feeding	*P* value
Duration of measurements, min	9.16 ± 1.55	9.15 ± 1.44	0.970	8.65 ± 1.38	7.82 ± 1.66	**0.0002**	8.50 ± 3.00	8.60 ± 3.04	0.796
Sucks, total	429.0 ± 143.7	343.0 ± 130.9	**0.0007**	353.5 ± 106.8	326.2 ± 110.8	**0.028**	425.1 ± 173.3	412.2 ± 192.6	0.522
Sucks/min	46.7 ± 12.9	37.9 ± 13.5	**0.0003**	41.1 ± 10.6	41.6 ± 10.7	0.577	50.6 ± 14.2	48.0 ± 15	0.094
Pauses, total	29.6 ± 12.8	31.4 ± 14.5	0.454	34.0 ± 14.4	26.2 ± 12.7	**<0.0001**	24.6 ± 16.7	20.7 ± 14.0	**0.018**
Pauses/min	3.2 ± 1.3	3.4 ± 1.4	0.457	3.9 ± 1.5	3.3 ± 1.3	**<0.0001**	2.8 ± 1.3	2.4 ± 1.2	**0.001**
Duration of pauses, total	169.6 ± 77.0	222.0 ± 114.2	**0.003**	213.8 ± 87.4	171.9 ± 79.4	**<0.0001**	161.7 ± 103.8	140.9 ± 116.2	0.115
Duration of pauses/min	18.6 ± 7.7	23.7 ± 11.0	**0.004**	24.4 ± 8.4	21.4 ± 8.3	**0.0007**	18.5 ± 9.0	15.9 ± 10.5	**0.018**

Data expressed as mean ± SD.

Maximal period of measurement was 10 minutes for the newborns of 21-28 days of age and 15 minutes for infants of 3-5 months of age.
